# Prognostic significance of occult lymph node metastases in breast cancer: a meta-analysis

**DOI:** 10.1186/s12885-021-08582-1

**Published:** 2021-07-30

**Authors:** Guixin Wang, Shuhao Zhang, Meiling Wang, Lin Liu, Yaqian Liu, Lianjun Tang, He Bai, Haidong Zhao

**Affiliations:** 1 General Surgery Department, Dalian University Affiliated Xinhua Hospital, Dalian, 116000 China; 2 Breast Surgery Department, The Second Hospital of Dalian Medical University, Dalian, 116000 China; 3 Cardiology Department, The First Hospital of Qinhuangdao, Qinhuangdao, 066000 China; 4 General Surgery Department, Dalian Jinzhou First People’s Hospital, Dalian, 116000 China

**Keywords:** Breast cancer, Occult metastases, Axillary lymph nodes, Survival, Recurrence

## Abstract

**Background:**

Occult metastases in axillary lymph nodes have been reported to be associated with poor prognosis in patients with breast cancer. However, studies on the prognostic value of occult metastases have shown controversial results. This meta-analysis aimed to evaluate the prognostic significance of occult lymph node metastases in breast cancer.

**Methods:**

Studies published until May, 2020, which retrospectively examined negative lymph nodes by stepsectioning and/or immunohistochemistry, were retrieved from MEDLINE, EMBASE, CNKI, and Cochrane Library databases. The pooled Relative Risk (RR) with 95% confidence interval (95% CI) for overall survival (OS) and disease-free survival (DFS) were calculated to examine the associations between occult metastases and prognosis.

**Results:**

Patients with occult metastases in axillary lymph nodes had poorer five-year DFS (RR = 0.930; 95% CI = 0.907–0.954) and OS (RR = 0.972; 95% CI = 0.954–0.990). Furthermore, the DFS (RR = 0.887; 95% CI = 0.810–0.972) and OS (RR = 0.896; 95% CI = 0.856–0.939) of patients with occult metastases were significantly lower after a ten-year follow-up.

**Conclusions:**

Occult metastases in the axillary lymph nodes are associated with poorer DFS andOS of patients with breast cancer. Occult metastases might serve as a predictive factor of survival outcomes in patients with breast cancer.

## Background

Axillary lymph node (ALN) status is an important prognostic indicator of survival in breast cancer [1]. In 1948, Saphir et al. showed that a limited number of sections from the axillary lymph nodes of patients with breast cancer are insufficient to detect metastases [2]. Since then, occult metastases have been defined as metastases that were not initially assessed, but detected by further examinations [3]. Over the following decades, multiple new techniques have been introduced to improve lymph node biopsy. Using step-sectioning and immunohistochemical (IHC) staining, occult metastases have been frequently detected in 12–23% of women with breast cancer, who initially exhibit negative axillary lymph nodes on hematoxylin and eosin (H&E) staining during routine pathological examination [4–10]. In addition, some studies also used reverse transcriptase-polymerase chain reaction (RT-PCR) to detect specific mRNA [6, 7, 11].

The prognostic significance of occult metastases remains controversial. Although several studies indicated occult metastases impacts OS or DFS [9, 12–15], others argued that occult metastases have no significant prognostic value [6, 10, 16, 17]. Furthermore, the routine use of IHC to stage lymph nodes has beenquestioned in recent large sample size studies. The National Surgical Adjuvant Breast and Bowel Project randomized controlled trial B-32 (NSABP B-32) indicated that occult metastases were an independent prognostic variable in survival, however, the difference in outcome at 5-years was small (1.2 percentage points) [14]. The American College of Surgeons Oncology Group (ACOSOG) Z0010 study also demonstrated that IHC evidence of occult metastases was not significantly associated with OS [18]. Hence, the current National Comprehensive Cancer Network (NCCN) guideline for breast cancer does not recommend routine IHC to define node involvement [19]. Although several systematic reviews have been published on the association between occult metastases and survival [3, 16], an update including recent studies is still necessary. This meta-analysis systematically evaluated the association between occult lymph node metastases and survival among patients with breast cancer.

## Methods

The present systematic review and meta-analysis was performed according to the recommendations of the Preferred Reporting Items for Systematic Reviews and Meta-Analyses (PRISMA) statement.

### Search strategy

The literature review was performed in PUBMED, EMBASE, China National Knowledge Infrastructure (CNKI), and Cochrane Library until May 1, 2020. The following search terms were used: (breast cancer OR breast carcinoma OR breast neoplasms), (lymph node OR lymph-node), (occult metastases OR micrometastases OR isolated tumor cells), and (prognosis OR prognostic OR survival OR survival rate OR survival analysis OR mortality OR recurrence). Relevant reviews, meta-analyses, and references cited in these papers were also checked for potential studies. Abstracts or unpublished reports were not considered. If more than one article was published by the same author using the same case series, the study with the highest number of subjects was selected. All the searches were conducted by two reviewers independently, and any disagreement was resolved through discussion.

### Inclusion and exclusion criteria

The following inclusion criteria were applied: ①evaluation of the association between occult metastases and survival outcome of breast cancer patients, ② case-control or cohort design, ③description of the survival outcomes of the studies in terms of disease-free or overall survival, and ④full texts based on original data. The exclusion criteria employed were: ①no control group (lymph node-negative group), ②lack of Kaplan-Meier methods or life-table analyses, and ③short follow-up period (< 5 years).

### Data extraction and study quality assessment

All data were extracted independently by two reviewers, according to pre-specified selection criteria. Disagreement was resolved by consensus and discussion with the third investigator. The following data were extracted: pathological assessment of the removed lymph nodes, number of control group, number of patients with lymph node occult metastases, tumor stage, follow-up period, performance of axillary lymph node dissection, disease-free/overall survival rates, administration of adjuvant systemic therapy, and results of multivariable analyses (Table 1). If the survival data were not provided in a table or text in the chosen articles, they would be extracted from the survival curves by Engauge Digitizer version 10.8 (GitHub, Open Source software). To prevent overlap of the data from studies that described subpopulations besides a total population, only subpopulations were taken into account for the calculation of relative risks. The Newcastle-Ottawa scale (NOS) was applied to assess the quality of the study [38].

**Table 1:** The main characteristics and quality scores of the included studies

Author (reference)	Year	PA	No. of patient		Stage	% AST		FU, y	Survival, % (OM vs pN0)				NOS
			OM	pN0		OM	pN0		5-y DFS	5-y OS	10-y DFS	10-y OS	
Fisher ER [20]	1978	SS (20 μm), H&E	19	59	I	0	0	5.1 a*	71 vs 68	–	–	–	8
Rosen PP [21]	1982	SS (48 μm), H&E	9	19	I	0	0	NR	89 vs 69	–	61 vs 62	–	7
Wilkinson E [22]	1982	SS (24–48 μm), H&E	89	436	NR	0	0	5 min	–	82 vs 80	–	64 vs 70	7
IBCSG [15] (no peri-op CT)	1990	SS (48 μm), H&E (6 levels)	55	555	I-II	0	0	5 med	61 vs 76	–	–	–	9
IBCSG [15] (peri-op CT)	1990	SS (48 μm), H&E (6 levels)	30	283	I-II	100	100	5 med	54 vs 68	–	–	–	9
Gelea MH [4]	1991	H&E + IHC (2 levels)	9	89	I-IIA	0	0	NR	–	100vs74	–	65 vs 62	7
de Mascarel [23]	1992	SS (1500 μm), H&E (1 level)	120	785	I-III	0	0	6.9 med	80 vs 88	89 vs 95	43 vs 78	61 vs 86	8
Elson CE [24]	1993	IHC (2 levels)	20	77	NR	0	0	5.7 a*	69 vs 71	83 vs 91	–	–	7
Hainworth PJ [25]	1993	IHC (1 level)	41	302	I-III	0	0	6.6 med	68 vs 84	87 vs 85	–	–	9
Nasser IA [26] (< 0.2 mm)	1993	SS (150 μm), H&E (5 levels) + IHC (1 level)	31	109	NR	0	0	11 a*	93 vs 81	–	78 vs 68	–	7
Nasser IA [26] (> 0.2 mm)	1993	SS (150 μm) H&E (5 levels) + IHC (1 level)	19	109	NR	0	0	11 a*	62 vs 81	–	51 vs 68	–	7
Tsuchiya A [27]	1996	IHC (3 levels)	3	182	NR	NR	NR	NR	100 vs 91	–	–	–	7
Clare SE [28]	1997	SS (150 μm) H&E + IHC (5 levels)	11	75	NR	0	0	6.7 med	71 vs 84	90 vs 95	–	–	7
Gerber B [29]	1997	H&E + IHC (2–6 levels)	18	141	I-IIA	68	100	4.3 a	70 vs 86	–	–	–	8
Cote RJ [30]	1999	IHC (1 level)	148	588	I-II	NR	NR	12 med	69 vs 74	–	55 vs 63	73 vs 78	8
Braun S [11]	2001	IHC (3 levels)	13	137	I-II	0	0	4 med	91 vs 83	91 vs 95	–	–	8
Cummings MC [31]	2002	SS (100 μm) H&E + IHC (4 levels)	53	150	NR	NR	NR	10.3 med	67 vs 86	83 vs 93	67 vs 82	75 vs 87	8
de Mascarel [32] (IDC)	2002	SS (1500 μm) H&E + IHC (1 level)	13	116	NR	0	0	24 med	84 vs 94	–	67 vs 89	–	8
de Mascarel [32](ILC)	2002	SS (1500 μm) H&E + IHC (1 level)	37	52	NR	0	0	18 med	91 vs 94	–	85 vs 87	–	8
Fisher ER [33]	2002	IHC (of original H&E)	63	213	I-II	100	100	9a*	–	88 vs 93	–	89 vs 87	8
Millis RR [6] (ITC)	2002	HE&IHC (1 level)	23	417	NR	0	0	13.2 (OM) 18.9 (pN0) med	–	83 vs 87	–	69 vs 77	8
Millis RR [6] (mi)	2002	HE&IHC (1 level)	57	417	NR	0	0	13.2 (OM) 18.9 (pN0) med	–	84 vs 87	–	78 vs 77	8
Umekita Y [13]	2002	IHC	21	127	NR	100	100	8.2 med	75 vs 95	86 vs 99	–	–	8
Gebauer G [34]	2003	examination SS (H&E 6 levels), followed by H&E + IHC (2 levels)	14	198	NR	0	0	NR	86 vs 88	85 vs 91	66 vs 84	72 vs 76	8
Reed W [7](ITC)	2004	IHC (1 level)	21	340	I-IIA	0	0	25.6 med	–	81 vs 91	75 vs 78	74 vs 84	8
Reed W [7] (mi)	2004	IHC (1 level)	16	340	I-IIA	0	0	25.6 med	–	80 vs 91	75 vs 78	75 vs 84	8
Kahn HJ [35]	2006	IHC (1 level)	29	175	NR	NR	NR	8 med	70 vs 77	89 vs 87	67 vs 69	79 vs 72	8
Marinho VF [36]	2006	IHC	26	162	NR	NR	NR	6.8 med	82 vs 90	78 vs 89	78 vs 78	69 vs 79	8
Querzoli P [12] (ITC)	2006	SS (100 μm) H&E (4 levels) + IHC (3 levels)	24	328	I-II	33.3	27	8 med	83 vs 95	–	–	–	8
Querzoli P [12] (mi)	2006	SS (100 μm) H&E (4 levels) + IHC (3 levels)	25	328	I-II	33.3	27	8 med	93 vs 95	–	–	–	8
Tan LK [8] (ITC)	2008	SS (50 μm) H&E + IHC (2 levels)	61	285	NR	0	0	17.6 med	77 vs 88	87 vs 92	68 vs 83	70 vs 80	8
Tan LK [8] (mi)	2008	SS (50 μm) H&E + IHC (2 levels)	17	285	NR	0	0	17.6 med	59 vs 88	94 vs 92	41 vs 83	59 vs 80	8
Loya A [10]	2009	H&E (1 level) + IHC (3 levels)	8	43	II-III	100	100	5.25 med	100 vs 88	100vs95	100 vs 88	100 vs 95	8
Park D [9] (ITC)	2009	SS (100 μm) H&E (2 levels) + IHC (10 levels)	53	200	NR	11.2	7.2	8.2 med	91 vs 94	–	–	–	8
Park D [9] (mi)	2009	SS (100 μm) H&E (2 levels) + IHC (10 levels)	31	200	NR	11.2	7.2	8.2 med	83 vs 94				8
Weaver DL [14]	2011	SS (500–1000 μm) H&E + IHC	616	3268	NR	NR	NR	7.9 med	86 vs 89	95 vs 96	–	–	8
Charles WK [37] (ITC)	2015	IHC	4657	81,693	I-IV	NR	NR	3.1med	–	92 vs 92	–	–	9
Charles WK [37] (mi)	2015	IHC	6720	81,693	I-IV	NR	NR	3.1med	–	88 vs 92	–	–	9

PA = pathological assessment of lymph nodes after original pathological assessment; AST = adjuvant systemic therapy; FU = follow up; DFS = disease-free survival; OS = overall survival; OM = occult breast cancer metastasis; MVA = multivariable analysis; IDC = invasive ductal carcinoma; ILC = invasive lobular carcinoma; ITC = isolated tumor cell ≤0.2 mm in diameter; mi = micrometastases from > 0.2 mm to ≤2 mm; H&E = hematoxylin and eosin staining; SS = step sectioning; IHC = immunohistochemical staining; NR = not reported; a* = average; min = minimum; med = median; NOS = Newcastle-Ottawa scale score

### Statistical analysis

The five- and ten-year relative risk (RR) of disease-free and overall survival was compared between the occult metastases group and control (lymph node negative) group. Statistical heterogeneity was measured using *I*^*2*^ (*I*^*2*^ > 50% was considered statistically significant heterogeneity). If significant heterogeneity was detected, the random-effects model was used and sensitivity analysis performed by removing one study at a time to calculate overall homogeneity and effect size. Otherwise, a fixed-effects model was employed [39]. Egger’s regression method was used statistically to assess publication bias (*p* <  0.05 was considered statistically significant) [40]. Statistical analysis was performed using STATA version 12.0 (StataCorp LLC, US), 95% confidence intervals (CIs) were reported, and *p* <  0.05 was considered statistically significant. All statistical tests were two-sided.

## Results

### Studies included in the meta-analysis

**Fig. 1:**
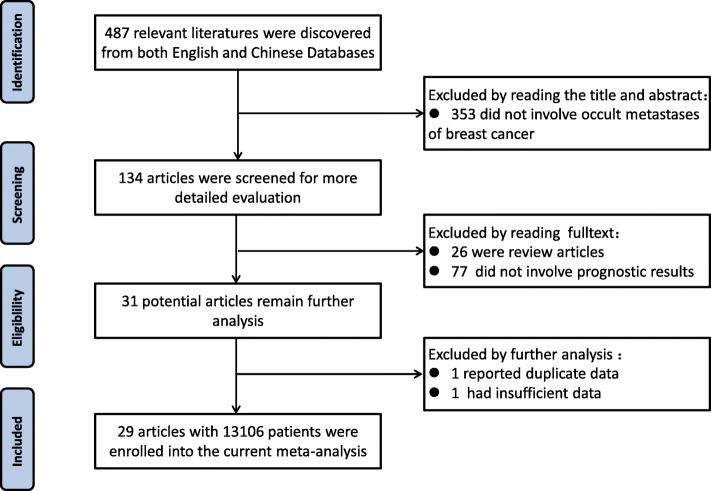
Flowchart of selection of studies and specific reasons for exclusion from the meta-analysis

A total of 487 papers were identified as relevant to the search words. After screening the title and reading the abstract, 134 articles were selected to for full text review, 26 studies were excluded as review or meta-analysis, and 77 articles were removed for not involving survival data or prognostic results. In further analysis of the remaining 31 potential articles, two articles were excluded: one reported duplicate data and the other one had insufficient data. Finally, 29 publications with 105,060 patients were included [4, 6–15, 20–37]. The flowchart of selection of studies and reasons for exclusion is presented in Fig. 1.

The characteristics and quality assessment results of the articles selected are summarized in Table 1. Of these, five articles only took step sectioning [15, 20–23], nine used step sectioning combined with immunohistochemical staining [8, 9, 12, 14, 26, 28, 31, 32, 34], four applied hematoxylin and eosin (H&E) staining with immunohistochemical staining [4, 6, 10, 29], while the rest only utilized immunohistochemical staining [7, 11, 13, 24, 25, 27, 30, 33, 35–37]. The breast cancer stage was described detailly in only 13 articles [4, 7, 10–12, 15, 20, 21, 23, 25, 29, 30, 33, 38], and follow-up duration ranged from 3.1 to 25.6 years. The use of adjuvant systemic therapy was not reported in 8 articles [14, 27, 28, 30, 31, 35–37], while it was applied to all or some patients in 7 articles [9, 10, 12, 13, 15, 29, 33], and none in the rest. Among the included articles, seven divided the patients with occult metastases into different subgroups [7–9, 12, 15, 26, 32]. As all subgroups analysis were regarded as separated studies, 36 studies were chosen for evaluation in this meta-analysis.

### Association between occult metastases and survival of patients

**Fig. 2:**
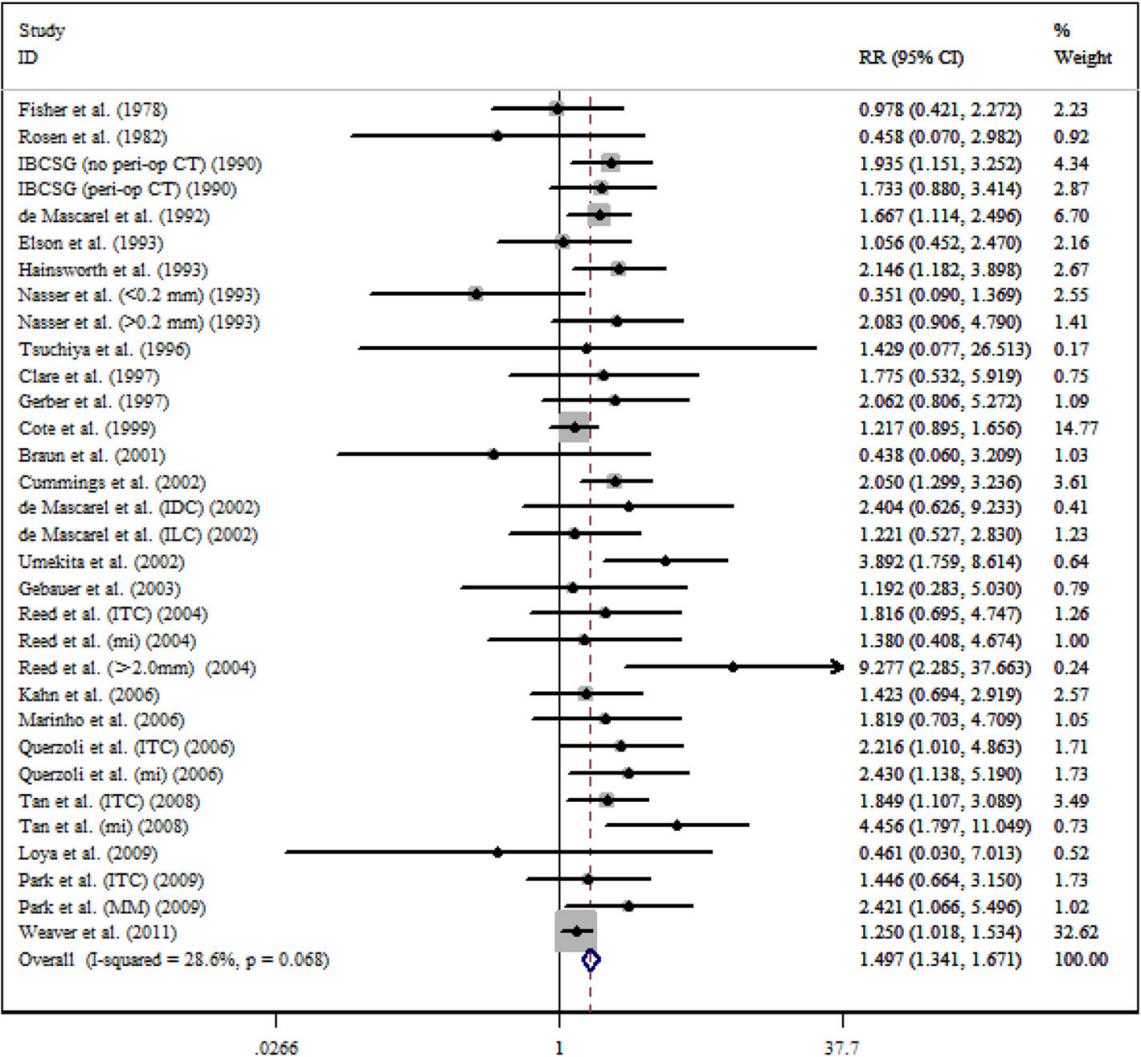
Association between 5-y DFS and the present of occult ALN metastasis

**Fig. 3:**
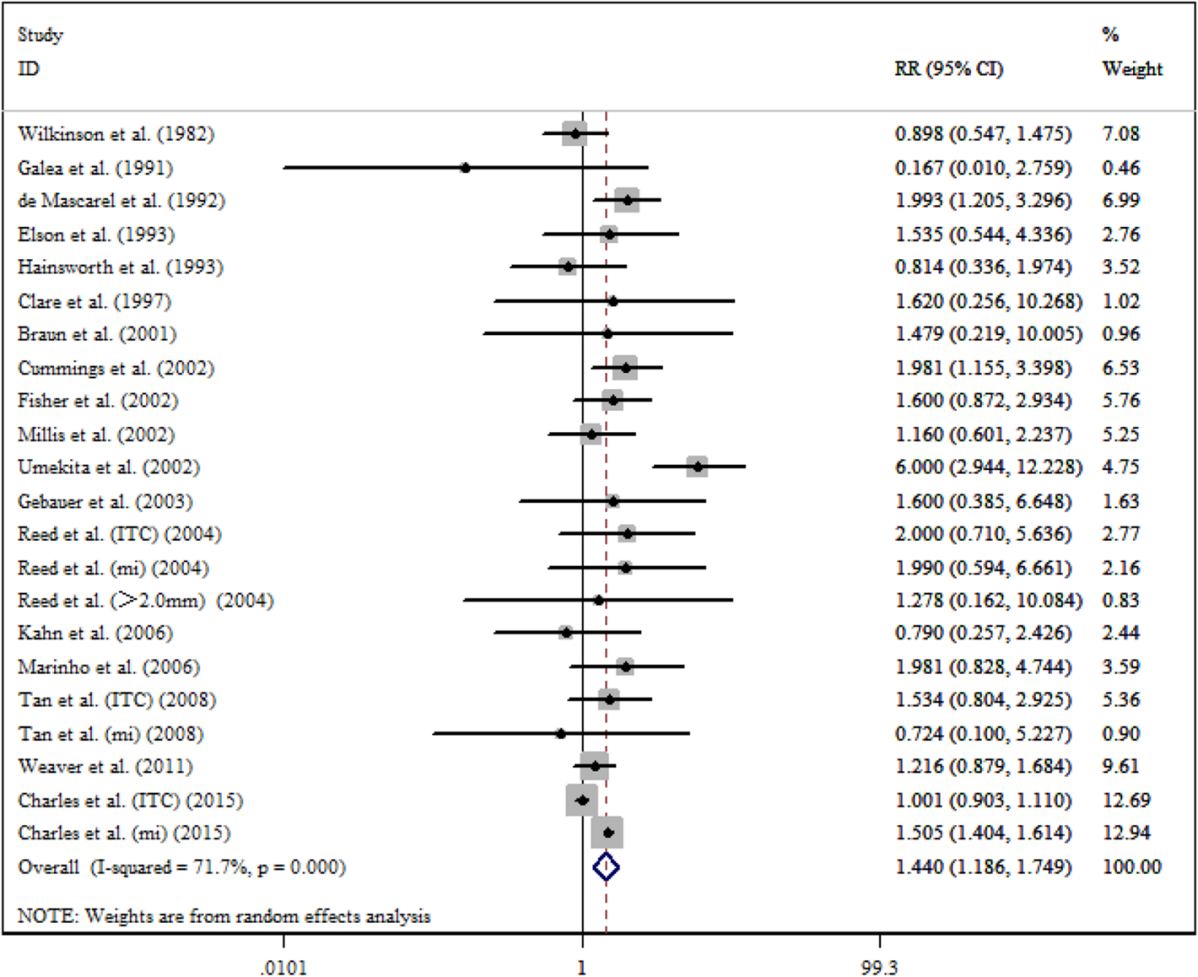
Association between 5-y OS and the present of occult ALN metastasis

**Fig. 4:**
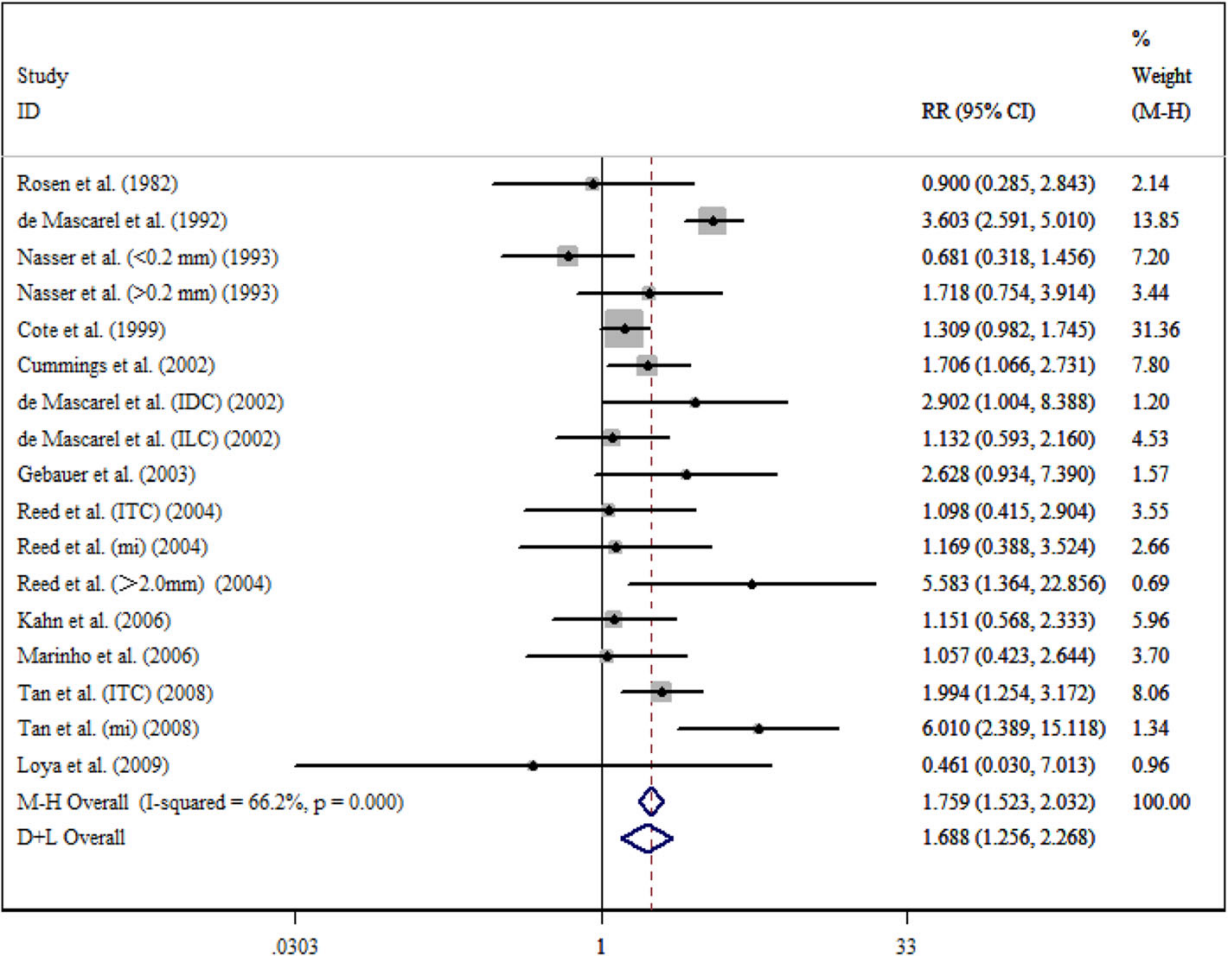
Association between 10-y DFS and the present of occult ALN metastasis

**Fig. 5:**
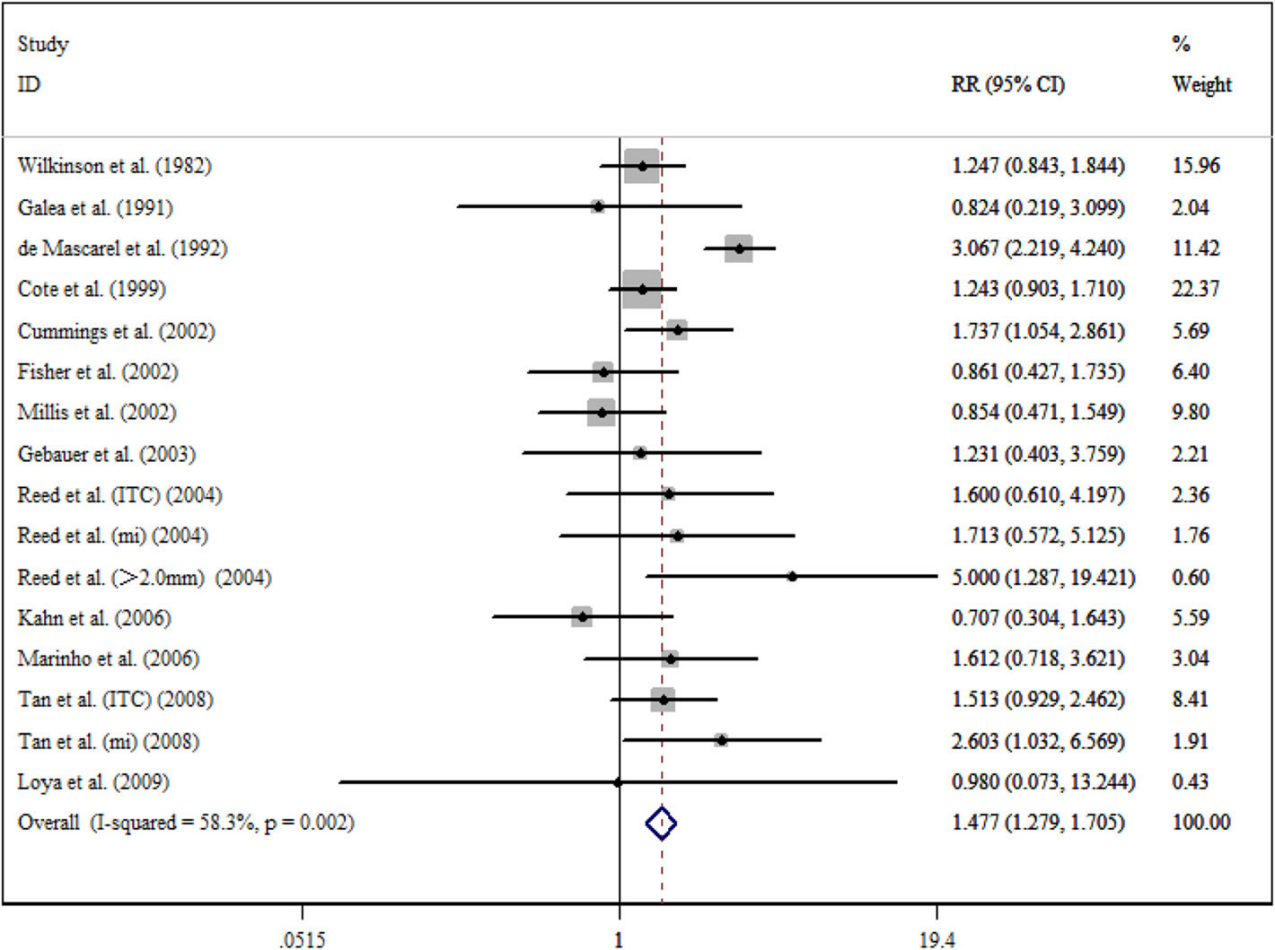
Association between 10-y OS and the present of occult ALN metastasis

**Fig. 6:**
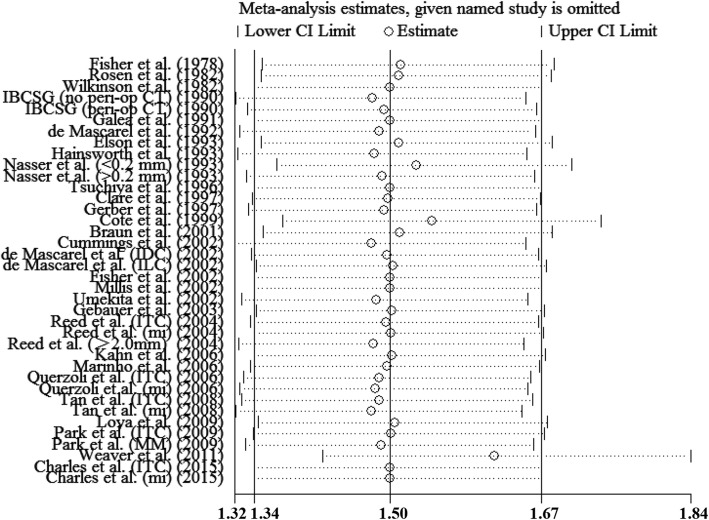
Results of the sensitivity analysis. Results when each study is excluded are shown by the point estimate of the HR and 95% CI

After a five-year follow-up, the results showed that occult metastases group was associated with poorer DFS (RR = 1.497; 95% CI = 1.341–1.671; *I*^*2*^ = 28.6%) (Fig. 2) and OS (RR = 1.440; 95% CI = 1.186–1.749; *I*^*2*^ = 71.7%) (Fig. 3). After a ten-year follow-up, the results also revealed poorer DFS (RR = 1.688; 95% CI = 1.256–2.268; *I*^*2*^ = 66.2%) (Fig. 4) and OS (RR = 1.477; 95% CI = 1.279–1.705; *I*^*2*^ = 58.3%) (Fig. 5) in patients with occult metastases. As obvious heterogeneity was observed in the study of 5-yr OS and 10-yr FDS, a random-effects model was utilized in these analysis. The results of the sensitivity analysis were consistent after excluding several studies [14, 30], with the confidence interval of RR not significantly decreasing (Fig. 6).

### Evaluation of publication bias

**Fig. 7:**
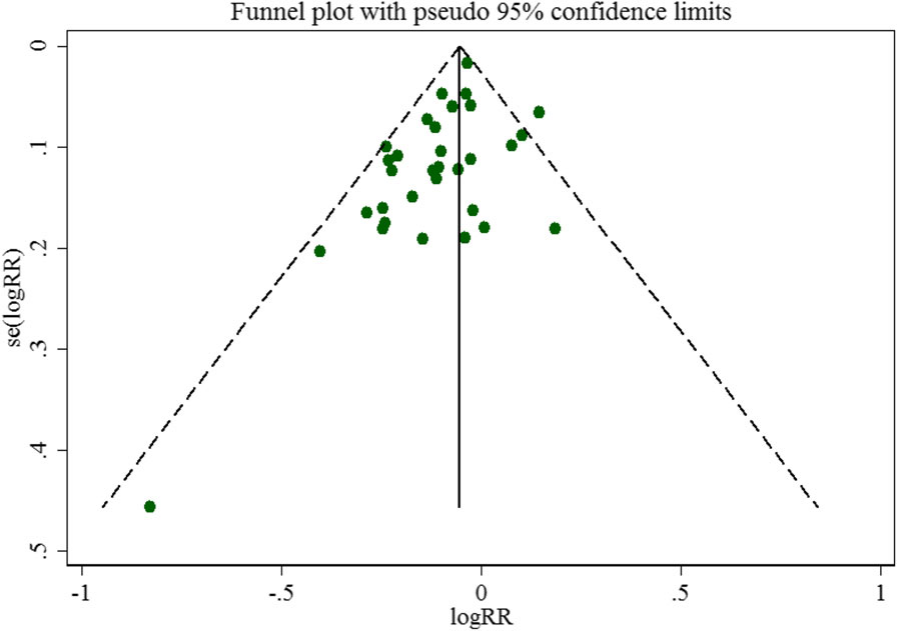
Funnel plot analysis on the detection of publication bias in the meta-analysis of Prognostic Significance of Occult Lymph Node Metastases in breast cancer

As including almost all studies, the studies of 5-yr DFS were conducted by using funnel plots and Egger’s test to assess publication bias. The funnel plot was approximately symmetrical (Fig. 7) and the result of Egger’s test (*P* = 0.567) revealed no obvious publication bias among the studies.

## Discussion

This meta-analysis of 36 studies aimed to explore the association between occult metastases in axillary lymph nodes and long-term prognosis ofpatients with breast cancer. In this meta-analysis, occult metastases were associated with worse DFSand OS than nagetive lymph nodes after 5-years and 10-years’ follow-up. These results demonstrated that occult metastases could be an independent prognostic factor in breast cancer patients with negative nodes on initial biopsy. Although the result of the sensitivity analysis were consistent, occult metastasis showed a worse prognosis after excluding the *NSABP B-32* study [14].. This heterogeneity might be due to the second largest sample size and the relatively smaller difference in 5-year survival. Although the included studies differed in terms of patient population, pathological assessment, follow-up duration, and methodology, their results were generally homogenous. This is not the first meta-analysis to evaluate the relationship between occult metastases and survival in patients with breast cancer, howevwe, it has several strengths over the previous meta-analysis [3]. The previous meta-analysis pointed out that presence of occult metastases was associated with poorer DFS and OS. This current meta-analysis included recent large sample size studies and obtained consistent results [14, 38].

The results were predominantly consistent with two previous large sample size studies, which albeit demonstrated that patients with occult micrometastases in axillary lymph nodes had a poorer survival [14, 37]. Weaver et al. [14] found that occult metastases were an independent prognostic factor in patients with sentinel lymph nodes (SLN) that were negative on initial examination. However, they indicated that additional evaluation, including IHC analysis, had no clinical benefit because the difference between survival was statistically significant relatively small. A larger study including 93,070 patients also demonstrated a difference in OS between patients with occult metastases and those with IHC-negative lymph nodes [37]. However, in further multivariate analysis in subgroups, micrometastases (0.2–2 mm diameter), rather than isolated tumor cells (< 0.2 mm diameter or < 200 cells, ITC), remained an independent predictor for survival.

The results of this meta-analysis differ from those of ACOSOG Z0010 study, which demonstrated no obvious difference in 5-year OS between occult metastases and no metastasis [18]. The difference might be due to that ACOSOG Z0010 was limited to early-stage T1 and T2 tumors. Several studies included in our analysis also reported that occult metastases could not predict a poorer survival in patients with breast cancer [6, 11, 20, 21, 32, 35]. This lack of significant difference might be due to the small sample size and the pathological examination techniques. It is worth mentioning that the Z0011 study has led to significant changes in the surgical treatment of breast cancer [41]. Fewer patients now receive further axillary surgery even in the presence of macrometassis. However, similar to the Z0010 study, the patients enrolled in the Z0011 study were also relatively at a lower risk: the tumor stage was T1–2, of which about 70% were T1 and 71% had only one positive SLN. Moreover, all patients underewnt breast-conservation surgery, and more than 96% of them received subsequent adjuvant systemic therapy. Therefore, the impact of occult metastasis on the survival of patients with a higher risk of recurrence (larger tumor size, mastectomy without adjuvant treatment, etc) needs further study.

The size of lymph node metastasis and subsequent treatment may affect the survival of patients with occult metastases. Weaver et al. [14] reported that the hazard ratio for death was 1.38 (95% CI = 1.02–1.87) in patients with isolated tumor cells and 1.91 (95% CI = 1.41–2.59) in those with micrometastases or macrometastases, when compared with patients in whom occult metastases were not detected. The MIRROR study showed that ITCs or micrometastases in SLN were associated with a lower 5-year DFS among patients who did not receive adjuvant therapy. Meanwhile, DFS was improved in patients with ITCs or micrometastases who received adjuvant therapy [42]. Consistent with the above results, this meta-analysis found that occult metastasis in axillary lymph nodes might affect the prognosis of patients, especially those who do not receive adjuvant therapy after surgery.

The relationship between axillary lymph node ITC/micrometastases and recurrence has not been fully established. Although studies have confirmed that adjuvant systemic therapy can improve the prognosis of patients, current evidence is still insufficient to support the replacement of axillary lymphadenectomy (ALND) with adjuvant systemic therapy alone. A further analysis of the MIRROR trial showed that patients with SLN micrometasis who did not undergo axillary treatment had an higher 5-year regional recurrence rate [43]. Therefore, we recommend to improve the detection rate of occult metastases in SLNs, especially for those patients who may be exempted from adjuvant therapy. In addition, adjuvant systemic therapy and axillary local radiotherapy should be performed in all patients with micrometastases, while ALND to minimize complications of surgery and improve quality of life while achieving a good prognosis.

The present analysis of the selected studies revealed that occult metastases could be detected in 9–42% of patients with breast cancer [4, 6–15, 20–37], mostly with micrometastases and ITCs, whereas macrometastases were inevitable. Hence, this study suggested that improvement of intraoperative assessment is necessary to increase the metastasis detection rate, especially macrometastases > 2 mm. In addition to intraoperative frozen section analysis, molecular techniques, such as RT-PCRand one-step nucleic acid amplification have been utilized, which could contribute to better detection rate of lymph node metastases, tumor staging, and subsequent therapeutic strategy [44]. The improvement of techniques can effectively improve the metastasis detection rate, but the impact on long-term survival requires further study.

The limitations of this study are as follows: (1) this meta-analysis was based on data from survival curves instead of pooled individual data; (2) pathological type, surgical options, adjuvant treatment regimen, and systemic therapy may be associated with DFS and OS, but these detailed data were not available in the majority of included studies or stratified analysis could not be conducted; (3) the majority of included studies had a retrospective design, so recall and selection biases may affect the results; (4) publication bias is an inevitable problem since this study is based on published articles, and ongoing or unpublished studies were not included in this meta-analysis.

## Conclusions

In summary, we found that occult metastases in the axillary lymph nodes of patients with breast cancer are an independent predictor of disease-free and overall survival. Moreover, it may indicate a relatively poor prognosis. However, because of non-standardized pathological examination and treatment, the prognostic value of occult metastases is still limited and further study is needed.

## Data Availability

All data generated or analysed during this study are included in this published article.

## Abbreviations

RR: relative risk
95% CI: 95% confidence interval
OS: overall survival
DFS: disease-free survival
ALN: axillary lymph node
IHC: immunohistochemical
RT-PCR: reverse transcriptase-polymerase chain reaction
ACOSOG: American College of Surgeons Oncology Group
NCCN: National Comprehensive Cancer Network
CNKI: China National Knowledge Infrastructure
NOS: Newcastle-Ottawa scale
H&E: hematoxylin and eosin
ITC: isolated tumor cells
ALND: axillary lymphadenectomy
SLN: sentinel lymph nodes

## References

[1] Fisher B, Bauer M, Wickerham DL, Redmond CK, Fisher ER, Cruz AB, et al. Relation of number of positive axillary nodes to the prognosis of patients with primary breast cancer. An NSABP update Cancer. 1983;52(9):1551–7. 10.1002/1097-0142(19831101)52:9<1551::aid-cncr2820520902>3.0.co;2-3. 10.1002/1097-0142(19831101)52:9<1551::aid-cncr2820520902>3.0.co;2-3 6352003

[2] Saphir O, Amromin GD (1948). Obscure axillary lymph node metastases in carcinoma of the breast. Proc Inst Med Chic.

[3] de Boer M, van Dijck JA, Bult P, Borm GF, Tjan-Heijnen VC (2010). Breast cancer prognosis and occult lymph node metastases, isolated tumor cells, and micrometastases. J Natl Cancer Inst.

[4] Galea MH, Athanassiou E, Bell J, Dilks B, Robertson JF, Elston CW (1991). Occult regional lymph node metastases from breast carcinoma: immunohistological detection with antibodies CAM 5.2 and NCRC-11. J Pathol.

[5] McGuckin MA, Cummings MC, Walsh MD, Hohn BG, Bennett IC, Wright RG (1996). Occult axillary node metastases in breast cancer: their detection and prognostic significance. Br J Cancer.

[6] Millis RR, Springall R, Lee AH, Ryder K, Rytina ER, Fentiman IS (2002). Occult axillary lymph node metastases are of no prognostic significance in breast cancer. Br J Cancer.

[7] Reed W, Bohler PJ, Sandstad B, Nesland JM (2004). Occult metastases in axillary lymph nodes as a predictor of survival in node-negative breast carcinoma with long-term follow-up. Breast J.

[8] Tan LK, Giri D, Hummer AJ, Panageas KS, Brogi E, Norton L, Hudis C, Borgen PI, Cody HS (2008). Occult axillary node metastases in breast cancer are prognostically significant: results in 368 node-negative patients with 20-year follow-up. J Clin Oncol.

[9] Park D, Karesen R, Naume B, Synnestvedt M, Beraki E, Sauer T (2009). The prognostic impact of occult nodal metastasis in early breast carcinoma. Breast Cancer Res Treat.

[10] Loya A, Guray M, Hennessy BT, Middleton LP, Buchholz TA, Valero V, Sahin AA (2009). Prognostic significance of occult axillary lymph node metastases after chemotherapy-induced pathologic complete response of cytologically proven axillary lymph node metastases from breast cancer. Cancer..

[11] Braun S, Cevatli BS, Assemi C, Janni W, Kentenich CR, Schindlbeck C (2001). Comparative analysis of micrometastasis to the bone marrow and lymph nodes of node-negative breast cancer patients receiving no adjuvant therapy. J Clin Oncol.

[12] Querzoli P, Pedriali M, Rinaldi R, Lombardi AR, Biganzoli E, Boracchi P, Ferretti S, Frasson C, Zanella C, Ghisellini S, Ambrogi F, Antolini L, Piantelli M, Iacobelli S, Marubini E, Alberti S, Nenci I (2006). Axillary lymph node nanometastases are prognostic factors for disease-free survival and metastatic relapse in breast cancer patients. Clin Cancer Res.

[13] Umekita Y, Ohi Y, Sagara Y, Yoshida H (2002). Clinical significance of occult micrometastases in axillary lymph nodes in "node-negative" breast cancer patients. Jpn J Cancer Res.

[14] Weaver DL, Ashikaga T, Krag DN, Skelly JM, Anderson SJ, Harlow SP, Julian TB, Mamounas EP, Wolmark N (2011). Effect of occult metastases on survival in node-negative breast cancer. N Engl J Med.

[15] Prognostic importance of occult axillary lymph node micrometastases from breast cancers. International (Ludwig) Breast Cancer Study Group. Lancet. 1990;335:1565–8. https://pubmed.ncbi.nlm.nih.gov/1972494/. 1972494

[16] Valachis A, Nearchou A, Polyzos NP, Lind P, Berqvist J. Occult metastasis in sentinel node: should this affect the clinical decision making? A systematic review and meta-analysis. Cancer Res. 2011;71:(24 Supplement):P3-07-34-P3-07-34.

[17] Takeshita T, Tsuda H, Moriya T, Yamasaki T, Asakawa H, Ueda S, Sato K, Aida S, Tamai S, Matsubara O, Hase K, Yamamoto J (2012). Clinical implications of occult metastases and isolated tumor cells in sentinel and non-sentinel lymph nodes in early breast cancer patients: serial step section analysis with long-term follow-up. Ann Surg Oncol.

[18] Giuliano AE, Hawes D, Ballman KV, Whitworth PW, Blumencranz PW, Reintgen DS, Morrow M, Leitch AM, Hunt KK, McCall LM, Abati A, Cote R (2011). Association of occult metastases in sentinel lymph nodes and bone marrow with survival among women with early-stage invasive breast cancer. JAMA..

[19] William JD BO, Jame A, Rebecca A, Kimberly HA, Sarah LB, Harold JB, Chau D. NCCN Clinical Practice Guidelines in Oncology: Breast Cancer. National Comprehensive Cancer Network; 2021. Available from: https://www.nccn.org/professionals/physician_gls/pdf/breast.pdf.

[20] Fisher ER, Swamidoss S, Lee CH, Rockette H, Redmond C, Fisher B. Detection and significance of occult axillary node metastases in patients with invasive breast cancer. Cancer. 1978;42(4):2025–31. 10.1002/1097-0142(197810)42:4<2025::AID-CNCR2820420452>3.0.CO;2-J. 10.1002/1097-0142(197810)42:4<2025::aid-cncr2820420452>3.0.co;2-j 213191

[21] Rosen PP, Saigo PE, Braun DW, Beattie EJ, Kinne DW (1982). Occult axillary lymph node metastases from breast cancers with intramammary lymphatic tumor emboli. Am J Surg Pathol.

[22] Wilkinson EJ, Hause LL, Hoffman RG, Kuzma JF, Rothwell DJ, Donegan WL (1982). Occult axillary lymph node metastases in invasive breast carcinoma: characteristics of the primary tumor and significance of the metastases. Pathol Annu.

[23] de Mascarel I, Bonichon F, Coindre JM, Trojani M (1992). Prognostic significance of breast cancer axillary lymph node micrometastases assessed by two special techniques: reevaluation with longer follow-up. Br J Cancer.

[24] Elson CE, Kufe D, Johnston WW (1993). Immunohistochemical detection and significance of axillary lymph node micrometastases in breast carcinoma. A study of 97 cases. Anal Quant Cytol Histol.

[25] Hainsworth PJ, Tjandra JJ, Stillwell RG, Machet D, Henderson MA, Rennie GC, McKenzie I, Bennett RC (1993). Detection and significance of occult metastases in node-negative breast cancer. Br J Surg.

[26] Nasser IA, Lee AK, Bosari S, Saganich R, Heatley G, Silverman ML (1993). Occult axillary lymph node metastases in "node-negative" breast carcinoma. Hum Pathol.

[27] Tsuchiya A, Sugano K, Kimijima I, Abe R (1996). Immunohistochemical evaluation of lymph node micrometastases from breast cancer. Acta Oncol.

[28] Clare SE, Sener SF, Wilkens W, Goldschmidt R, Merkel D, Winchester DJ (1997). Prognostic significance of occult lymph node metastases in node-negative breast cancer. Ann Surg Oncol.

[29] Gerber B, Krause A, Reimer T (1997). Immunohistochemically detected lymph-node micrometastases in breast cancer and their correlation with prognostic factors. Breast Journal.

[30] Cote RJ, Peterson HF, Chaiwun B, Gelber RD, Goldhirsch A, Castiglione-Gertsch M (1999). Role of immunohistochemical detection of lymph-node metastases in management of breast cancer. Int Breast Cancer Stud Group Lancet.

[31] Cummings MC, Walsh MD, Hohn BG, Bennett IC, Wright RG, McGuckin MA (2002). Occult axillary lymph node metastases in breast cancer do matter: results of 10-year survival analysis. Am J Surg Pathol.

[32] de Mascarel I, MacGrogan G, Picot V, Mathoulin-Pelissier S (2002). Prognostic significance of immunohistochemically detected breast cancer node metastases in 218 patients. Br J Cancer.

[33] Fisher ER, Wang J, Bryant J, Fisher B, Mamounas E, Wolmark N (2002). Pathobiology of preoperative chemotherapy: findings from the National Surgical Adjuvant Breast and bowel (NSABP) protocol B-18. Cancer..

[34] Gebauer G, Fehm T, Merkle E, Jaeger W, Mitze M (2003). Micrometastases in axillary lymph nodes and bone marrow of lymph node-negative breast cancer patients--prognostic relevance after 10 years. Anticancer Res.

[35] Kahn HJ, Hanna WM, Chapman JA, Trudeau ME, Lickley HL, Mobbs BG (2006). Biological significance of occult micrometastases in histologically negative axillary lymph nodes in breast cancer patients using the recent American joint committee on Cancer breast cancer staging system. Breast J.

[36] Marinho VF, Zagury MS, Caldeira LG, Gobbi H (2006). Relationship between histologic features of primary breast carcinomas and axillary lymph node micrometastases: detection and prognostic significance. Appl Immunohistochem Mol Morphol.

[37] Kimbrough CW, McMasters KM, Quillo A, Ajkay N (2015). Occult metastases in node-negative breast cancer: a surveillance, epidemiology, and end results-based analysis. Surgery..

[38] Stang A (2010). Critical evaluation of the Newcastle-Ottawa scale for the assessment of the quality of nonrandomized studies in meta-analyses. Eur J Epidemiol.

[39] Higgins JP, Thompson SG, Deeks JJ, Altman DG (2003). Measuring inconsistency in meta-analyses. BMJ..

[40] Egger M, Davey Smith G, Schneider M, Minder C (1997). Bias in meta-analysis detected by a simple, graphical test. BMJ..

[41] Armando EG, Karla B, Linda M, Peter B, Pat WW, Peter B (2016). Locoregional recurrence after sentinel lymph node dissection with or without axillary dissection in patients with sentinel lymph node metastases: long-term follow-up from the American College of Surgeons oncology group (Alliance) ACOSOG Z0011 randomized trial. Ann Surg.

[42] de Boer M, Deurzen CH, Dijck JA (2009). Micrometastases or isolated tumor cells and the outcome of breast cancer. N Engl J Med.

[43] Manon JP, Maaike B, Peter B, Jos AD, Carolien HD, Marian BM (2012). Regional recurrence in breast Cancer patients with sentinel node micrometastases and isolated tumor cells. Ann Surg.

[44] Layfield DM, Agrawal A, Roche H, Cutress RI (2011). Intraoperative assessment of sentinel lymph nodes in breast cancer. Br J Surg.

